# Role of the Skin Prick Test in Urticaria Patients

**DOI:** 10.7759/cureus.21818

**Published:** 2022-02-01

**Authors:** Samruddhi Lote, Sanjeev B Gupta, Divya Poulose, Mahendra Singh Deora, Aditi Mahajan, Jaya Madhurya Gogineni, Sujay Saxena, Biswajit Chaklader

**Affiliations:** 1 Dermatology, Dr. D. Y. Patil Medical College, Hospital and Research Centre, Pune, IND; 2 Department of Radiology, Maharaja Yeshwantrao Hospital, Indore, IND; 3 Preventive Medicine, Dr. D. Y. Patil Medical College, Hospital and Research Centre, Pune, IND

**Keywords:** diagnosis, allergy test, allergens, spt, ige-mediated, urticaria

## Abstract

Background

Urticaria, a vascular reaction of the skin, is marked by the transient appearance of erythematous papules or plaques (wheals) of varying sizes that are blanchable and associated with severe pruritus which lasts from a few hours to days. The etiological factors for urticaria include food, drugs, bacterial foci, pollen, fungi, dust, worms, physical stimuli, stress, anxiety, insect stings, etc. Skin prick tests (SPTs) represent the cheapest and most effective method to diagnose immunoglobulin E-mediated type 1 allergic reactions such as urticaria. A history suggestive of clinical sensitivity supported by a positive test strongly implicates the allergen in the disease process. In this study, we aimed to detect the common allergens and correlate the findings of SPTs with various epidemiological characteristics of urticaria patients.

Methodology

A total of 100 patients with urticaria were included in this study. After receiving written and informed consent from patients, SPTs using a battery of 45 allergens were performed.

Results

In our study, SPT positivity was seen in 88 (88%) patients. The highest sensitization was noted toward *Dermatophagoides pteronyssinus* (house dust mite) (30%), followed by *D. farinae*, *Cynodon dactylon*, and peanuts (each comprising 24%), and *Ailanthus excelsa* (20%).

Conclusions

Finding the causative allergen in urticaria is often a difficult and long-drawn process, both for the physician and the patient. Our study identified an allergen in 88% of patients with urticaria, thereby showing that the SPT is a cost-effective, easy, and reliable tool for diagnosing and guiding treatments in urticaria patients.

## Introduction

Urticaria is a vascular reaction of the skin marked by the appearance of transient, smooth, slightly elevated, erythematous papules or plaques (wheals) of varying sizes which are blanchable and associated with severe pruritus which lasts from a few hours to days. Although the lesions are often self‑limiting and resolve without scarring, they may recur over weeks to months [1]. Urticaria occurs throughout the world, presenting with a lifetime occurrence of 8.8% [2].

Urticaria is referred to as acute when the symptoms are present for less than six weeks and chronic when symptoms persist for more than six weeks. Factors precipitating acute urticaria have been reported in approximately 50% of the cases. Some common factors include viral infections, food, medications, and insect sting reactions. Association with other infectious organisms such as those causing cystitis and tonsillitis has also been reported. Ingestion of food additives such as colorings (azo and non-azo dyes), anti-oxidants, preservatives (nitrates and nitrites), and aspartame (an artificial sweetener) can also cause urticaria [3,4].

Finding the causative allergen of urticaria is often a difficult and long-drawn process, both for the physician and the patient. Skin prick tests (SPTs) are performed by depositing drops of allergens on the lower forearm and reading the results after 15 minutes. SPTs are the cheapest and most effective method to diagnose urticaria. Positive skin tests with a history suggestive of clinical sensitivity strongly incriminate the allergen as a contributor to the disease process [5].

Hence, in this study, we aimed to investigate the causative allergens in patients with urticaria attending the outpatient department of our institute using SPTs and correlate the findings with various epidemiological features.

## Materials and methods

A total of 100 patients with urticaria were recruited for this study from September 2019 to August 2021. Patients aged 10 years and above with a clinical diagnosis of urticaria were included in this study. Pregnant and breastfeeding females, patients suffering from other cutaneous diseases, those with a history of anaphylaxis, and those with an active infection at the site of the SPT were excluded. Sociodemographic, clinical history, and written and informed consent were obtained from the patients.

**Figure 1:**
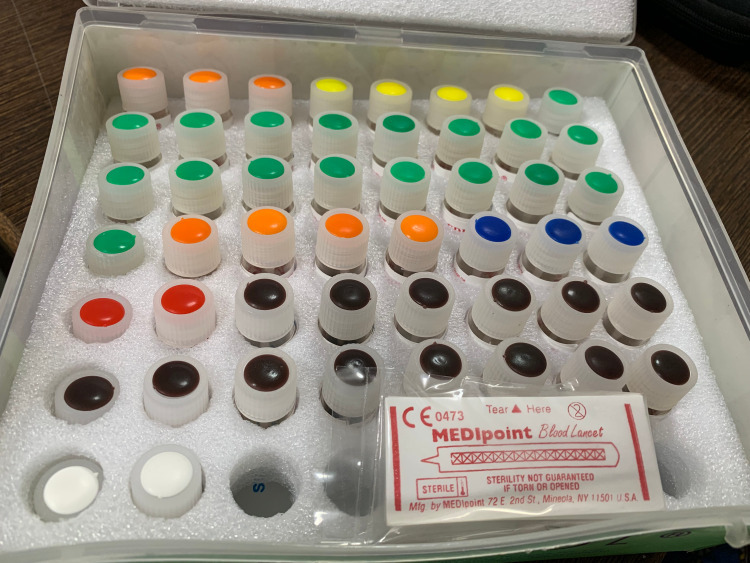
Set of allergens used in the study.

Patients were asked to discontinue drugs such as antihistamines and corticosteroids for three and fourteen days prior to the test, respectively. Mast cell stabilizers and tricyclic antidepressants were stopped one week prior to the test. Patients were instructed to abstain from applying any topical steroids and immunomodulatory creams to the area where the test would be performed one week prior to the test. The test was done on an area of the skin that was devoid of any lesions. The volar aspect of the forearms is the location most commonly used for performing the test. Histamine phosphate 10 mg/mL (positive control) and buffered saline in glycerol (negative control) were applied first. Thereafter, a total of 45 allergens were applied to each of the patients’ forearms [5] (Figure 1).

**Figure 2:**
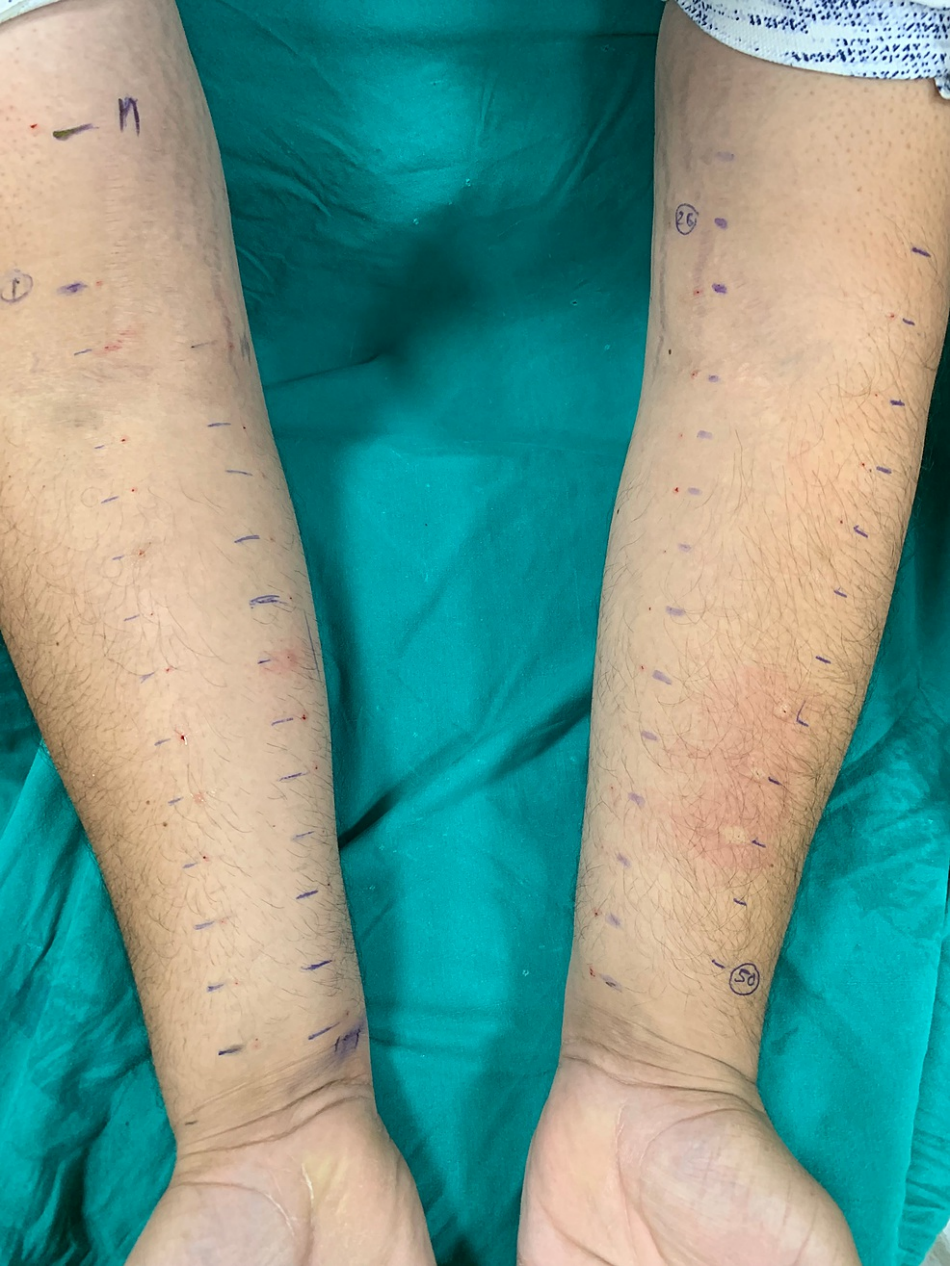
Skin prick test positivity seen in a patient.

Measures for resuscitation in case of anaphylaxis were kept handy. A droplet of each antigen was placed on cleaned forearms at 2-3 cm intervals, followed by puncturing with a lancet held at an angle of 90 degrees to prevent oozing of blood from the site. The results were calculated using a standard ruler after 15 minutes. A positive and negative result was seen with positive and negative controls, respectively. Positive results were those with a wheal diameter of 3 mm or more [5] (Figure 2).

A proforma in English and the native language Marathi was used to record all the findings. The results were subjected to statistical analysis using SPSS software thereafter (IBM Corp., Armonk, NY, USA) [5].

## Results

Out of the 100 patients recruited in our study, the number of males and females was equal. The most common allergens in females were *Dermatophagoides pteronyssinus* (28%) and *Ailanthus* (28%), whereas most males were sensitive to *D. pteronyssinus* alone (32%). The age of the participants ranged from nine to 57 years, with the maximum number of patients in the 31-45-year age group (39%); the mean age of the patients was 30.92 years. There were 12 (12%) patients in the 0-15-year age group, 38 (38%) in the 16-30-year age group, and 11 (11%) in the 46-60-year age group (Table 1).

**Table 1:** Age distribution of patients with urticaria.

Age (years)	Number of patients
0–15	12 (12%)
16–30	38 (38%)
31–45	39 (39%)
46–60	11 (11%)

In total, 18 (18%) patients had acute urticaria, while 82 (82%) had chronic urticaria. Furthermore, 53 (53%) patients gave a history of angioedema, and 26.41% of the patients were sensitive to both *D. pteronyssinus* and mushrooms.

**Figure 3:**
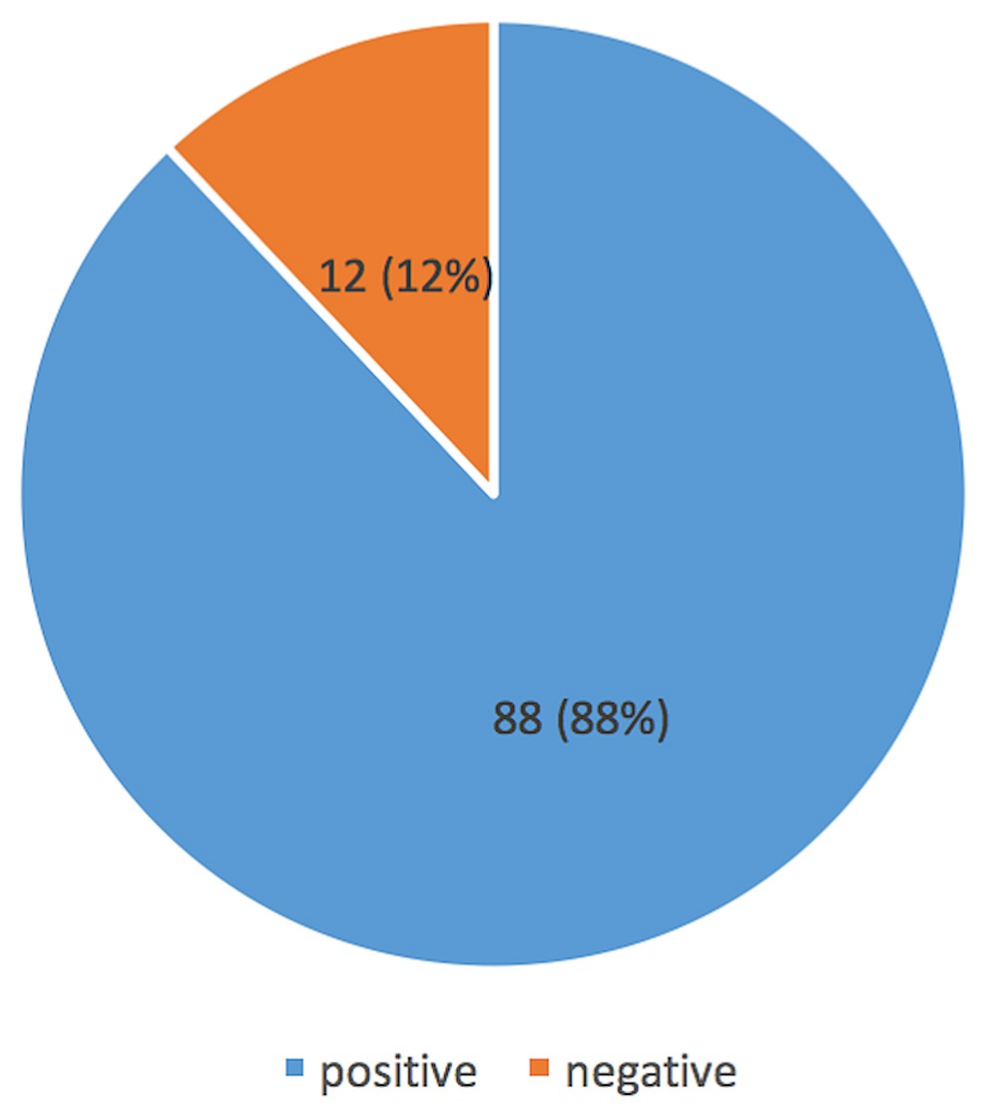
Reactivity to the SPT. SPT: skin prick test

A positive family history of atopic diathesis was seen in 18 (18%) of the patients, while the remaining 82 (82%) had no significant history. These patients were in the age group of 22-45 years with a male:female ratio of 1:2. Nine (50%) patients with a positive family history were allergic to mushrooms. The SPT was positive in 88 (88%) patients (Figure 3). In total, 54 (54%) patients showed positivity to 1-5 allergens, 31 (31%) patients to 6-10 allergens, three (3%) patients to 11-15 allergens, and 12(12%) patients did not show sensitivity to any of the allergens (Table 2).

**Table 2:** Number of allergens positive on the SPT. SPT: skin prick test

Number of allergens	Number of patients
0	12 (12%)
1–5	54 (54%)
6–10	31 (31%)
11–15	3 (3%)

**Figure 4:**
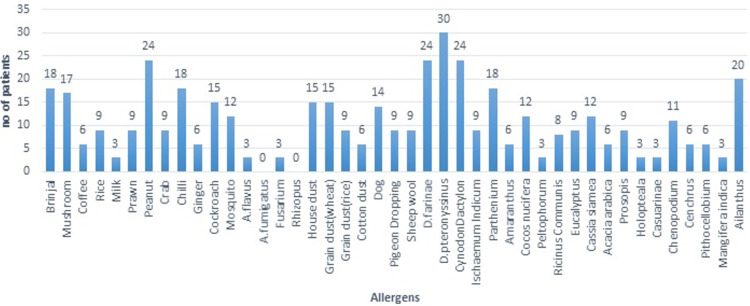
Skin sensitivity to various allergens.

The highest sensitization was seen to *D. pteronyssinus* (house dust mite) (30%), followed by *D. farinae*, *Cynodon dactylon*, and peanuts (each comprising 24%), and *Ailanthus excelsa* (20%). Among food allergens, peanut (24%) was the most common, followed by brinjal (18%), chili (18%), and mushroom (17%). *Aspergillus flavus *(3%) and *Fusarium* (3%) were the most common fungi showing positivity on the SPT. Dog epithelia (14%) was the most common epithelial allergen, followed by pigeon droppings (9%) and sheep wool (9%). The most common insect to show positivity on the SPT was cockroach (15%), followed by mosquito (12%). House dust (15%) and grain dust (15%) were the most common dust allergens. *D. pteronyssinus* was the most common mite to show sensitivity on the SPT, with 30 patients showing a positive result, followed by *D. farinae* (24%). Among the 18 pollens tested, *Cynodon dactylon* (24%) was the most common, followed by *Ailanthus* (20%) and *Parthenium* (18%) (Figure 4).

## Discussion

The SPT is a reliable tool in patients with urticaria as many patients show improvement after eliminating the allergen against which a positive result is observed. In this study, most patients with urticaria were in the 31-45-year age group (39%), which is comparable to the study by Sreejith et al. [6] where most patients (29.9%) were in the 33-42-year age group.

The gender distribution in our study was equal, with the male-to-female ratio of 1:1. In comparison, in studies by Sreejith et al. [6], Nath et al. [7], and Rebello et al. [8], the number of females was more than males. However, in the study by Mounika et al. [9], the male-to-female ratio was 1:0.8.

In this study, 53% of patients gave a history of angioedema compared to the study by Sreejith et al. [6], in which 33.8% of patients gave a positive history. In our study, the SPT was positive in 88 (88%) patients, which is comparable to the study by Rebello et al. [8] (82.5%). However, other studies by Bains et al. [5], Sreejith et al. [6], and Kulthanan et al. [10] reported the prevalence of positive SPT to be 63.4%, 68.8%, and 34.9%, respectively.

In this study, 54 (54%) patients had positivity to 1-5 allergens, 31 (31%) patients were positive to 6-10 allergens, and 3 (3%) patients were positive to 11-15 allergens. In comparison, Sreejith et al. [6] showed that 17 patients developed a positive reaction to only a single allergen (22.1%), 36 were positive for two or more allergens, and there was only a single patient who was positive for seven allergens. Caliskaner et al. [11] performed the test in 259 patients with chronic urticaria. In their study, 71 (27.4%) patients tested positive for one or more antigens.

The highest sensitization in our study was noted toward *D. pteronyssinus *(house dust mite) (30%) followed by *D. farinae* (house dust mite), *Cynodon dactylon* (pollen), peanuts (food) (each comprising 24%), and *Ailanthus excelsa* (pollen) (20%), which can be compared to the study by Caliskaner et al. [11], wherein the most common allergen was house dust mite (24.7%), followed by pollen (7.7%), cockroach (0.8%), and mold (0.4%). Tezcan et al. [12] reported that 3% of the patients were allergic to *D. farinae*, 42% to *D. pteronyssinus*, and 54% to grass pollen. Mahesh et al. [13] reported that 64% of patients had skin sensitivity to house dust mites.

Our study had some limitations. First, the panel of aeroallergens was selected based on the endemicity of the allergens to the city in which this study was conducted. Second, because this was a single-center study, a convenient sample was obtained.

## Conclusions

A causative allergen could be identified in 88% of our study participants. House dust mite (*D. pteronyssinus*) was found to be the most common allergen, followed by *Cynodon dactylon* (pollen), peanut, and *Ailanthus excelsa* (pollen). Hence, our findings show a possible association of urticaria with mite, pollen, and food, suggesting that the SPT can be a reliable tool for determining these allergens, and can assist clinicians in managing this condition by avoiding exposure to these allergens and preventing unnecessary exclusion by patients, especially from their diets.

## References

[1] Gaur SN, Bhati G (2015). Urticaria: an overview. Indian J Allergy Asthma Immunol.

[2] Zuberbier T, Balke M, Worm M, Edenharter G, Maurer M (2010). Epidemiology of urticaria: a representative cross-sectional population survey. Clin Exp Dermatol.

[3] Deacock SJ (2008). An approach to the patient with urticaria. Clin Exp Immunol.

[4] Folci M, Ramponi G, Brunetta E (2021). A comprehensive approach to urticaria: from clinical presentation to modern biological treatments through pathogenesis. Adv Exp Med Biol.

[5] Bains P, Dogra A (2015). Skin prick test in patients with chronic allergic skin disorders. Indian J Dermatol.

[6] Shreejith AP, George AE, Mathew R (2020). Skin prick test in chronic urticaria in a tertiary care centre in South India. J Med Sci Clin Res.

[7] Nath A, Balaji A, Thappa DM (2007). Prick testing in chronic idiopathic urticaria: a report from a tertiary care centre in south India. Internet J Dermatol.

[8] Rebello SM, Bhatt R, Sukumar D, Alapatt GF (2015). A study of skin prick in patients with chronic urticaria. Int J Recent Trends Sci Technol.

[9] Mounika K, Shivaswamy K (2017). Skin prick test positivity to house dust mites (HDM) in patients with chronic urticaria. Int J Res Dermatol.

[10] Kulthanan K, Wachirakaphan C (2008). Prevalence and clinical characteristics of chronic urticaria and positive skin prick testing to mites. Acta Derm Venereol.

[11] Caliskaner Z, Ozturk S, Turan M, Karaayvaz M (2004). Skin test positivity to aeroallergens in the patients with chronic urticaria without allergic respiratory disease. J Investig Allergol Clin Immunol.

[12] Tezcan D, Uzuner N, Sule Turgut C, Karaman O, Köse S (2003). Retrospective evaluation of epidermal skin prick tests in patients living in Aegean region. Allergol Immunopathol (Madr).

[13] Mahesh PA, Kushalappa PA, Holla AD, Vedanthan PK (2005). House dust mite sensitivity is a factor in chronic urticaria. Indian J Dermatol Venereol Leprol.

